# Kidney Xenotransplantation: Are We Ready for Prime Time?

**DOI:** 10.1007/s11934-023-01156-7

**Published:** 2023-04-22

**Authors:** Rafael Miyashiro Nunes dos Santos

**Affiliations:** Schiff Center for Liver Diseases, Miami Transplant Institute, University of Miami, 1801 NW 9Th Ave, Room 329, Miami, FL 33136 USA

**Keywords:** Kidney xenotransplantation, Xenotransplantation, Zoonoses, Xenoantigen, Genetic engineering, Genetic-engineered animals

## Abstract

**Purpose of Review:**

With the exponential increase in interest and great strides toward clinical application, many experts believe we are ready for kidney xenotransplant human trials. In this review, we will examine the obstacles overcome and those yet to be conquered, discussing the human trials performed and the questions they raised. Additionally, we will revisit overlooked aspects that may be crucial for improvements and suggest future approaches for xenotransplant research.

**Recent Findings:**

Improving survival in pig-to-non-human-primate models with the identification of an ideal immunosuppression regimen led to 3 cases of kidney xenotransplant in brain-dead humans with limited follow-up and a single clinical case of pig-to-human heart xenotransplant with 2-month survival.

**Summary:**

With limited human results and unlimited potential, xenotransplantation shines a beacon of hope for a brighter future. However, we must navigate through the complexities of balancing scientific progress and patient welfare, avoiding being blinded by xenotransplantation’s unquestionable potential.

## Introduction

Organ failure is the main cause of mortality in most population groups [1], and transplantation is the only cure for this ailment. In spite of this, only 10% of the global demand of organ transplants are been fulfilled according to the World Health Organization [2].

To decrease the kidney waiting list time, morbidity, and mortality, several strategies such as paired donation [3], donation after cardiac death [4], complex anatomy living donors [5], high KDPI kidneys [6], and ABO-incompatible renal transplantation [7] have been implemented. Unfortunately, this increase in yearly transplants has been outweighed by an even greater number of patients added to the waitlist, increasing from about 17,000 in 1988 to about 69,000 in 2022. This imbalance is projected to get even bigger, with the expected prevalence of end-stage kidney disease increasing 29–68%, from 690,000 to 971,000 and 1,259,000 by 2030 [8].

With this growing unmet need, longer waiting times and mortality are anticipated. This makes obvious the need of new therapeutic options for those patients.

Xenotransplantation has the potential to eliminate the waitlist completely. Recently, enthusiasm for this new therapeutic modality has reached its peak after human brain-dead kidney xenotransplants [9••] and later the first genetically modified pig-to-human heart xenotransplantation in a patient not a candidate for regular allotransplantation [10••].

With the exponential increase in interest and great strides toward clinical application, many experts believe we are ready for kidney xenotransplant human trials [11–13]. In this review, we will examine the obstacles overcome and those yet to be conquered, discussing the limited human trials performed and the questions they raised. Lastly, we will revisit overlooked perspectives that may aid in comprehending the clinical outcomes of transgenic animals and suggest different approaches for xenotransplant research.

## Pig as the Source Animal

Even though there was a relative clinical success with non-human-primates transplants in the 1960s [14], when the interest in xenotransplantation reemerged because of the long waiting list for conventional allotransplantation, scientists quickly realized that it would be impossible to source enough organs from those animals. Consequently, pigs were chosen as the best option due to their unlimited availability, similar anatomy and physiology to humans, and lower risk of zoonosis [15].

Nevertheless, due to its increased evolutionary distance to humans, new obstacles were added before clinical success could be achieved.

## Barriers to Xenotransplantation

### Xenoantigens

Genetic mutations during evolution have caused humans to lose function on some genes that are still functional in pigs. Some of these mutations lead to the presence of antigens in pigs that humans have pre-formed antibodies against. The most important, leading to hyperacute rejection and organ destruction in minutes to hours, is α1,3 galactosyltransferase (GGTA1) gene, that adds residues of Galactose-α 1,3-Galactose (α-Gal) to glycoprotein and glycolipids [16].

The first genetically engineered modalities to reduce that burden were the random incorporation of human complement-regulatory proteins (CD55 [17, 18], CD46 [19], and CD59[20]) in pig cells, which prevented the complement cascade progression and minimized the cellular damage.

In recent times, the field has undergone a profound evolution with the advent of targeted endonucleases (enzymes that can identify and cut a particular sequence of DNA such as zinc-finger nucleases [21], TALENS [22], and more recently CRISPR’s [23]) granting the capacity to selectively disrupt any gene of choice.

Other major xenoantigens that contribute to acute humoral xenograft rejection are Neu5Gc and Sda antigen. The former (Neu5GC) is produced by hydroxylation of Neu5Ac by the gene cytidine monophosphate-N-acetylneuraminic acid hydroxylase (CMAH) which is not present in humans but is present in pigs and old world monkeys [24]. Sda describes the blood group of the same name, produced by the gene Beta-1,4-N-Acetyl-galactosaminyltransferase 2 (β4GALNT2) [25].

Knockout of GGTA-1, CMAH, and β4GALNT2 genes on pigs results in organs with negative crossmatch in many patients waiting for kidney transplant [26•].

Interestingly, because old-world monkeys still have active CMAH gene, triple knockout pigs have increased IgM and IgG reactivity when compared to GGTA-1/B4GALNT2 knockout pigs. Consequently, the ideal pig for non-human-primate studies is different than for human clinical trials [27].

Other demonstrated xenoantigens with less acute repercussions are pig SLA class I and class II that can cross-react with human HLA class I and class II respectively [28–30].

### Incompatibility of Pig and Human Proteins

To date, several enzymes/proteins that have a molecular incompatibility between pigs and humans have been identified, most remarkably in the coagulation system.

Pig thrombomodulin, an endothelial transmembrane anticoagulant, can bind to human thrombin, but this complex fails to activate protein C [31, 32], leading to graft thrombosis.

Another well-known incompatibility leading to increase thrombosis is that pig tissue factor pathway inhibitor (TFPI) fails to inhibit blood coagulation initiated by human tissue factor [33].

Pig von Willebrand Factor can aggregate human platelets spontaneously, by binding to the GP1b receptor, leading to the post-reperfusion thrombocytopenia [34].

Other non-vital incompatibilities related to kidney xenotransplantation that still need further research are as follows:

- Renin-angiotensin system potentially leads to hypovolemia syndrome in non-human primates in pre-clinical trials [35].
- Pig erythropoietin has a similarity of 82% with its human counterpart [36], but it is uncertain if it works effectively in humans [37].
- Anti-diuretic hormone action may differ from pig to human due to different locations of the pig collecting ducts which can lower xenograft urine osmolality [38].

In relation to its function, pig kidneys can maintain most electrolytes within normal range after xenotransplantation, except for slightly elevated calcium level and lower range for phosphorus [38, 39].

The growth potential of pig organs exceeds that of their human counterparts. This could be a potential problem after xenotransplantation, especially in heart transplants where space is more restricted. This issue can be addressed by utilizing smaller size pig breeds (miniature pigs) or growth hormone receptor knockout. The latter will cause clinical features of Laron syndrome in the animals, which features include obesity, shorter stature with smaller bones, longer lifespan, lower cancer incidence, higher insulin sensitivity, reduction in fertility, juvenile hypoglycemia, and disproportionally small liver and kidneys [40].

### Cellular and Antibody-Antibody-Mediated Xenograft Rejection

Should hyperacute rejection be averted, the innate and adaptive immune system may trigger cellular and antibody-mediated xenograft rejection, leading to graft loss within mere days to weeks, unless sufficient immunosuppression is maintained [41].

Human CD8 + T cells can recognize pig SLA-I and cause direct cytotoxic damage to pig endothelium. Also, T cells can be activated by porcine (direct pathway [42]) and human (indirect pathway [43]) antigen-presenting cells (APC) through the interaction of T-cell receptor (TCR) and SLA/HLA class I and II coupled with costimulatory signals, such as CD40-154 and CD80-CD28 [44].

Despite the existence of healthy SLA class I knockout pigs [45], its effectiveness have not been extensively tested on pre-clinical trials in non-human primates, possibly not providing benefits [46•]. This is also true to pigs that express negative costimulatory signals [47, 48]. Since pig endothelial cells work as APC in xenotransplantation [49, 50], alternatives such as these could lead to diminished necessity for systemic immunosuppression, particularly a solution that would reduce SLA Class II expression [51].

On the other hand, medical therapies have undergone extensive testing in non-human-primate models. Regular immunosuppression protocols are not enough to avoid xenograft rejection after pig-to-non-human primates. Studies have shown that CD4 + depletion associated with blockage of CD40/CD154 pathway is the most effective regimen with significant survival increase in non-human-primate models [52•]. Unfortunately, anti-CD154 antibodies have been associated with thrombogenic complications, and anti-CD40 antibodies are not yet approved for use in organ transplantation by the Food and Drug Administration (FDA) [53].

Additionally, anticomplement therapy has been shown to increase survival [54••]. This could be associated with a lack of transgene expression [46•] in some studies where this was shown to be true.

### Zoonosis

One of the biggest concerns in xenotransplantation is the transmission of new pathogens to humans. Recent events have shown the health and socioeconomic problems associated with the dissemination of new infectious diseases.

Most of porcine pathogens can be eradicated by non-infected heard selection, captivity in germ-free facilities with infectious diseases screening protocols, isolation of animals from exterior environment, vaccinations, elective sterile C-section for birth with early weaning, and control of food source [55].

Nevertheless, porcine endogenous retroviruses (PERVs), which are integrated into multiple copies (up to 95 per cell [56]) in every pig genome, cannot be prevented by these isolation measures.

While there are available options for the prevention [57, 58] and treatment [59], there is no consensus if complete inactivation of PERV by genetic engineering is necessary [60, 61]. Even though transmission from pig-to-human cells have been demonstrated in vitro under certain conditions, over 200 pig-to-non-human primates and over 200 clinical pig-to-human xenotransplant procedures (islet cell, ex vivo perfusion with pig livers or spleens, neuronal cell transplant) were performed without PERV transmission [55].

## Current Best Practices in Kidney Xenotransplantation

The cost of xenotransplantation research renders it nearly impossible to create controlled pig-to-non-human-primate trials with direct comparison of each successive genetic modification and diverse medications.

Despite these limitations, some statistically significant conclusions can be drawn from the available data [54••]:

- Triple knockout (GGTA1, CMAH, B4GALNT2) is the baseline genetic modification
- Transgenic animals for complement regulatory proteins (CD55, CD46 or CD59) significantly and statistically increase survival
- Utilization of anti-CD40 antibody increases survival
- Utilization of anticomplement therapy (CVF or anti-C5) increases survival

Other genetic modifications are probably beneficial for xenotransplantation, but its presence or absence was not directly evaluated by using the same immunosuppression in kidney xenotransplantation. One example that probably has a benefit is transgenic pigs for human thrombomodulin, since this modification has shown increased survival benefit in pig-to-non-human-primate heart xenotransplantation [61].

In terms of kidney xenotransplantation survival in non-human-primate models, there are a couple of series with long survival. In the first series, animals were GGTA-KO/hCD55 transgenic and did not require anticomplement therapy, with mean survival > 265 days in 9 animals (some animals were still alive at the time of publication) [52•]. Another series with double or triple knockout and no transgenes required anticomplement therapy for increased survival with a mean > 263 days in 7 animals (one animal alive at publication) [46•].

Apparently, transgene for complement-regulatory sequences has survival benefit and may require less immunosuppression after xenotransplant (medication not FDA-approved). Perhaps an even greater survival benefit would be achieved if all kidney cells expressed them (to be discussed in the next section).

## Genetic Engineering Obstacles to Overcome

One factor that is not emphasized as much as it should be is the deficiencies of current genetic engineering protocols.

**Fig. 1:**
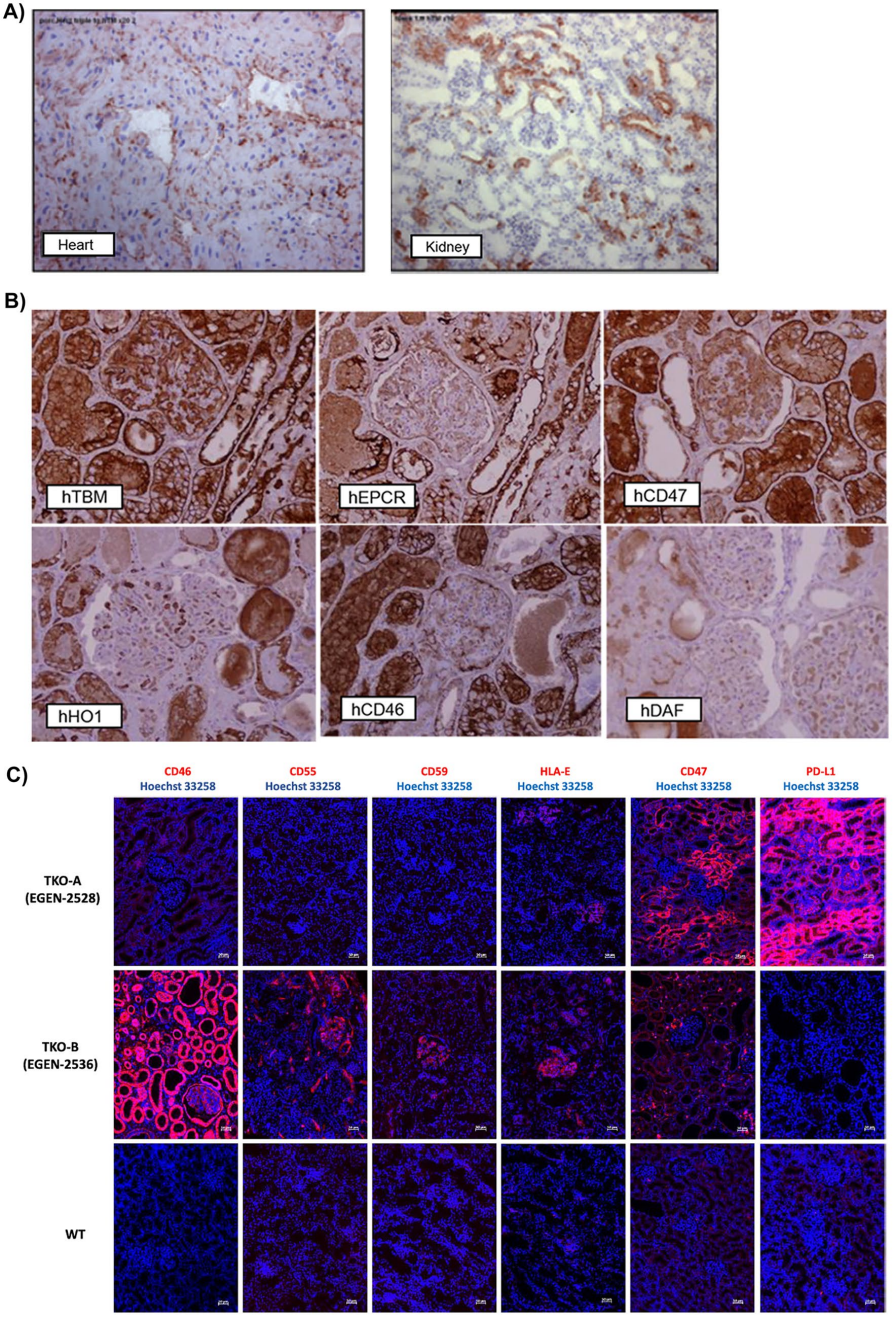
Variation of transgene expression in pigs. Immunohistochemistry in **A** and **B** with brown stating representing pig cells with human transgene. Kidney immunofluorescence in **C** with pink staining representing human transgene expression. **A** Difference in expression of human thrombomodulin in the heart and kidneys from a single pig. Reused and modified with permission from [32]. **B** Expression level of each of the six transgenes in the same kidney on the 10-GE pig transplanted in a brain-dead human. Reused and modified with permission from [78••]. **C** Difference in expression of different human transgenes in pig TKO-A and TKO-B. Pig A was considered positive for CD46, CD55, and CD59 but with low expression and had high expression of HLA-E and CD47. Pig B was considered to have high expression of CD46, CD55, and CD59 and moderate expression of HLA-E and CD47. PD-L1 was only added to pig A and expressed in a high amount. Reused with permission from [48]

There are several problems with most gain of function pig protocols. The organs present patchy expression of the transgene, sometimes with organs not presenting any expression whatsoever. Figure 1 shows the variation in transgene expression in different organs in the same animal and different transgenes in the same organ.

In addition to that, the original pig gene is often, if not always, maintained when its removal would be beneficial. This leads to a competition for receptors resulting in lower function of the human gene. A protocol that removed the pig gene showed consistently high function of the human counterpart [62]. Another example of competition for receptors resulting in less function of both proteins is the creation of pigs that express a non-functional copy of CIITA, resulting in competition with the functioning copy and less SLA class II expression in pigs [51].

Moreover, due to the utilization of exogenous promoters to drive transgene expression, choosing the right one becomes very difficult.

Also, these exogenous promoters are susceptible to loss of function over time due to epigenetic factors, leading to a decrease of human gene expression progressively.

Lastly, there is a lack of transparency about the gene expression profile and promoters utilized in several studies [63]. Even in the first clinical case of pig-to-human heart xenotransplantation, there is no information about the gene expression profile of the 6 transgenes present in the heart, which promoters were used, how many copies of the genes were present, data about possible expression loss with aging (from prior genetically identical pigs) [10••]. Transparency and documentation of these data can help understand some of the clinical complications and should be commonplace in xenotransplantation research. Drug trials always have the active compound dosage recorded; xenotransplantation should strive for the same.

Here are direct quotes about the problems of genetic engineering in pigs. Those quotes help us understand the importance of the characterization of each transgene expression profile in each experiment:

“The variable expression of transgenes, both within pig tissues and among individual pigs, will pose considerable challenges in producing a consistent xenograft for transplantation in humans”[9••]. Dr. Montgomery justifying selecting pigs without transgenes for the kidney xenotransplant performed in brain dead humans.

“Initially thought to be expressing human thrombomodulin, but later found not to be expressing this human coagulation-regulatory protein [61].” After a pig-to-non-human-primate kidney xenotransplant and realizing that the expected transgene was not present in the organ.

“Although hHO-1 transgenic pigs were cloned from the same donor cell line, differences in the expression levels of hHO-1 between clones could be detected [64].” Pigs were genetically identical, however presented very different phenotypes.

“Human TFPI should have been constitutively expressed in the surface of the endothelial cells (pig). However, expression of TFPI was minimal in most cases [65].”

“These data indicate that the transgene expression level decreases with aging in vivo [66].”

“The ICAM-2 promoter was clearly superior in expression/functionality of TBM in endothelial cell (…) when compared with endothelial cells from pTBM-TBM pigs. However, immunochemistry of heart tissue indicated more robust expression of hTBM generated by the porcine endogenous TBM promoter [67].” Authors reveal dissimilar outcomes in different organs by choosing different promoters.

“High expression of CD46 in all pigs and of CD55 (in Group A) on pAECS was documented, with variable expression of TMB (in group B) from 8 to 96% … No attempt was made to determine the expression of the transgenes in the explanted hearts [68].” Authors acknowledging that there is a vast difference in results by utilizing different techniques to create transgenic animals, but organ gene expression is not always verified in the experiments. This could cloud the interpretation of results.

“For clinical xenotransplantation, miniature swine could be used, rather than GHRKO pigs derived from domestic pigs (…) it might be easier to inhibit the growth of these pigs with GHRKO than begin or continue to genetically engineer miniature swine [40]”. Author comments how important is to keep the current transgenic pigs, risking side effects of growth hormone blockage, since starting a new transgenic pig line with a more suitable size would require extensive screening for the pigs with better transgene expression profile.

To avoid variable gene expression and the requirement for cell clone screening, we at the Miami Transplant Institute in collaboration with the Schiff Center for Liver Diseases are researching a new concept of algorithmic gene targeting protocol with initial cell line success. This protocol should create identical pigs eliminating the need for pig screening and increase human transgene function.

## Regulatory, Legal, and Ethical Concerns

Despite the formidable genetic obstacles posed by current protocols, regulatory, legal, and ethical considerations may pose an even greater challenge for xenotransplant implementation.

Many animal rights organizations are against the use of animals for research, including xenotransplantation.

Others argue that scientists may disturb the fundamental nature of a species, despite the fact that selective breeding has caused more extensive modifications than the ten genetic alterations engineered for xenotransplantation [69].

As xenotransplant may lead to zoonotic diseases with public health implications, one of the most challenging aspects of its implementation is the possible requirement of life-long monitoring for patients and their close contacts [70]. This concern has some regulatory agencies, such as the Council of Europe Recommendations Rec (2003) 10 of the Committee of Ministers to Member States on xenotransplantation, Article 21, stating that patients may have to waiver some of their human rights for close follow-up and monitoring.

Regulatory and legal aspects will require worldwide cooperation for the initiation and growth of xenotransplant. Owing to the pervasive effects of globalization and unequal availability of healthcare and regulatory policies across regions, individuals seeking xenotransplantation may resort to traveling to countries with looser policies for the life-saving procedure, imposing increased risks for zoonosis upon their return home where animal husbandry and organ screening regulations are more stringent [71].

Moreover, there are questions if a collective consent should be required prior to initiation of xenotransplant trials since this procedure may carry risks for a broader population beyond the patients participating in the study [71].

## Solid Organ Xenotransplantation in Humans

In late 2021, Dr. Montgomery and his team on NYU performed a xenotransplant between pig-to-brain-dead-human recipient with initial apparent success followed months later by another case [9••]. The pigs of choice were alpha-1,3-galactosyltransferase knockout, and the kidney grafts were prepared 2 months prior to transplantation to incorporate a porcine thymus implanted under the renal capsule [72]. Standard immunosuppression was used, and heparin drip was added for systemic anticoagulation due to the lack of anti-thrombotic transgenes. The duration of the protocol was 54 h.

Experts [73] criticize this work due to the chosen genetic background. Other points against the study were that immunosuppression was not the considered gold standard for xenotransplantation (lack of CD154-CD40 co-stimulatory blockage) [48, 52•, 68, 74–77] and the fact that native nephrectomy was not performed, so the real function of the xenografts could not be evaluated properly, even though they produced more urine than the native kidneys in both cases.

Shortly after, a pig heart to a patient that was not a candidate for allotransplantation (compliance issues) was performed. The FDA approved the experimental procedure since the outcome was not expected to be worse than the alternative (lifelong ECMO). The pig genetic background was a 10-gene-edit (4 knockouts and 6 human transgenes) and immunosuppression added complement C1 esterase inhibitor and humanized anti-CD40 monoclonal antibody (not approved by the FDA for allotransplantation) to the standard protocol [10••].

Patient had a good early clinical outcome with extubation on the second day of transplant, and ECMO was discontinued on the 4th day. Soon after, patient acquired unusual infections for a heart transplant patient such as peritonitis for *Escherichia coli* and *Candida tropicalis* and later developed porcine CMV viremia despite negative porcine CMV screening in the donor. Later, patient developed antibody-mediated rejection with 40% myocyte necrosis, and life support was discontinued on the 60th day due to irreversible xenograft damage.

The last trial was a pig-to-brain-dead-human kidney xenotransplantation performed at the University of Alabama [78••]. The pig kidney had the same genetic background from the heart patient [10••], and the immunosuppression used was the same as in allotransplantation associated with a continuous dose of heparin. After native bilateral nephrectomy, the right and left 10-GE pig kidneys were transplanted separately into the recipient.

Unfortunately, this case did not perform well. There was minimal urine output from the left and less than 1L in 3 days for the right kidney with increasing serum creatinine and BUN (over 6 mg/dL and 150 mg/dL respectively). Also, histologic findings 24 h after xenotransplantation were consistent with thrombotic microangiopathy, even with pigs with 2 genes to avoid such complication in addition to the continuous heparin infusion.

These recent human trial experiments left us with questions:

- What is the best infection screening protocol for those animals, since the established method failed in preventing zoonosis in the only clinical case?
- Could genetic manipulations that minimize rejection such as SLA class I or even SLA class II knockout decrease the immunosuppression burden? Could it have prevented the rejection in the heart patient? Could negative costimulatory transgenic pigs have helped?
- Why did Alabama’s kidney xenograft fail since the beginning, demonstrated by extremely low urine output and progressive increase in BUN and creatinine associated with thrombotic microangiopathy features?Could the brain-dead status alone be sufficient to justify the outcome? Should we perform more brain-dead cases with more stable recipients to determine if that was the case?
  - Could the standard immunosuppression be insufficient and responsible for those issues?
  - Could an eventual previous vascular arteriosclerosis with endothelial damage in the recipient be responsible for some of the findings (57yo brain-dead recipient with BMI 35.2 as opposed to healthy young non-human primates)? If so, how will be the results in patients with even more vascular damage waiting for kidney transplantation?
  - Is an eventual unevenness of human transgenic expression in the pig organ a possible factor in the development of thrombotic microangiopathy?
  - Is the presence of pig thrombomodulin responsible for the lack of function of the human counterpart due to competition for the receptor between the two?
- Will patients require full anticoagulation for life after xenotransplantation?
- Will patients require more immunosuppression than the standard FDA-approved medication for allotransplantation? If so, will that cause more infections, as evidenced in the heart transplant patient and in the animal models [54••]? In that case, would xenotransplant be less morbid than dialysis to consider its clinical application?
- Could xenotransplantation cause HLA sensitization and preclude patients to go to allotransplantation later?
- Should we answer these questions in a real clinical trial or should we have them answered prior to submit patients stable on dialysis through this new promising surgical procedure?

## Option for Kidney Xenotransplant Research

As mentioned prior, old-world monkeys are not a perfect model for xenotransplant research because they have higher positivity in crossmatch to pigs when compared to humans. Also, because they still have a functional CMAH gene and humans don’t, the ideal pig for non-human-primate research would be different than the one utilized in human clinical trials [27].

For those reasons, many researchers believe that advancement will occur quicker when clinical trials start [11, 12].

One alternative option that could answer the remaining questions without risk for patients stable on dialysis is to increase the observation time of brain-dead recipients after kidney xenotransplantation. The current experiments lasted no longer than 3 days [9••, 78••].

There is an anecdotal case report of a patient that was brain-dead and supported for over 20 years [79, 80]. This was not the only case, there was another case of a patient that was supported for 165 days before ventilation was discontinued [81].

Anecdotal cases could be the exception, and no realistic survival would be expected in any significant number of cases. However, there is a clinical scenario that proves that maintaining a brain-dead patient for many days is realistic.

In pregnant patients, one systematic review with 19 individually reported cases, 2 of them were supported for over 100 days with an average of 38.3 days (2–107). In 12 (63%) of 19 reported cases, it was possible to support the patient until delivery of a viable child, and in 4 cases, there was intra-uterine death with the survival of the mother [82].

## Conclusion

Xenotransplant presents a shining beacon of hope for the future of humankind, yet we must not let its potential blind us to the complexities of balancing scientific progress and patient welfare.

Due to the lack of availability of organs for all patients, transplant is the only specialty that a new therapeutic option with worse outcomes than the gold standard would ever be considered. With this in mind, we need to establish where we should draw the line of acceptable risk for our patients.

If we consider the kidney xenograft as a bridge to allotransplantation, clearly it would be ethical when:

- xenotransplant morbimortality becomes lower than those of patients on the waitlist.
- xenotransplant does not preclude, impair or delay allotransplantation due to sensitization.

Unfortunately, it is known that HLA-sensitized patients can have antibodies against SLA. One would wonder if the opposite were true as well. Eventually, this could be tested by performing longer-term brain-dead trials.

Regarding waiting list mortality, authors are considering xenotransplantation for patients that are at least 1 year on dialysis and, for optimal outcomes after xenotransplant, suggest that patients must be between 55 and 60 years old, no more than 65, without diabetes or other important comorbidities, no prior sensitization for HLA (could increase risk of SLA cross-reactivity) [83]. Other authors suggest using the kidney transplant decision aid for the selection of patients for xenotransplantation [84].

Additionally, the animals considered for human kidney xenotransplant trials are either knockout only, requiring 2 non-FDA approved drugs for transplantation in addition to the standard immunosuppression, or transgenic pigs with uneven human gene expression that would benefit from more reliable genetic engineering protocols, as seen in Fig. 1.

Table 1 contrasts the waiting list and kidney xenotransplant current results. When comparing both, it is difficult to defend the latter without reservations. Other authors agree with this assessment [85, 86].

**Table 1:** Matched kidney wait list and xenotransplant risk comparison

Waiting list	Xenotransplant
5-year cumulative incidence of mortality 16.3%*	2-year mortality rate of 100% in non-human-primate models
25% death or too sick for transplant in 3 years**	Primary graft non-function in human brain-dead recipient wit 10 GE pig
	Added non-FDA approved immunosuppression requirement
	Uncertainty of sensitization to allotransplantation
	Zoonosis in the only clinical case due to failed screening protocol
	Uncertainty of isolation protocols for patients, medical providers and family members
	Uncertainty of follow-up regimen and the ability to withdraw consent from protocol due to public health hazard concerns

^*^From the US Renal Data System (https://usrds-adr.niddk.nih.gov/2022/end-stage-renal-disease/7-transplantation), patients aged 45–64 years old

^**^From Kidney Transplant Decision Aid (https://www.srtr.org/tools/kidney-transplant-decision-aid/). Worse outcome variables selected (male, white, and O blood group) for a patient weighing 200 lbs and 5 feet 9 inches high, excluding comorbidities to match initial candidates for xenotransplant trial (diabetes, high PRA, history of cancer and peripheral artery disease) in the worse performing kidney program in the USA with at least 5 kidney transplants in the period. Accessed in January/2023

Conversely, if we consider a patient with acute liver failure who does not have a clinical indication for allotransplantation (actively drinking, non-compliant), mortality can reach close to 100% without transplantation in certain conditions. Perhaps this would be the best candidate for xenotransplantation today [87]. A protocol of ex vivo perfusion could be enough to support the patient while the native organ recovers. In one report, 4 patients were submitted to ex vivo perfusion of unmodified pig livers for acute liver failure. One was stabilized for 10 days and bridged successfully for liver allotransplantation [88]. Another report bridged successfully 2 of 2 patients in acute liver failure with transgenic hCD55/hCD59 pig livers [89].

Additionally, any patient waiting for a heart transplant in the same clinic scenario faced by the team in Maryland would also unquestionably benefit from xenotransplantation [10••].

As we stand on the brink of new times, with one foot in the present and one in the future, we should not rush blindly forward. Instead, let us remember the enduring principles embodied in the oath we all swore, to guide us as we navigate the uncertain and ensure that we build a bridge sturdy enough to cross. If we use those principles as our compass, we can rest assured that we will reach our destination without losing our way.

Just as a reminder, the shortened version of the Hippocratic Oath proclaimed at Johns Hopkins University School of Medicine goes as follows:

“I do solemnly swear, by that which I hold most sacred, that I will be fully committed to those I serve, to the utmost of my power, holding myself aloof from wrong, from corruption, and from the tempting of others to vice, that I will exercise my art, solely for the care of my patients, and will give no drug, and perform no operation, without justifiable purpose, nor ever suggest it. And in proportion, as I am faithful to this, my oath, may happiness and good repute be ever mine. The opposite if I shall be forsworn.”
